# Supplementary material for: Study on modular smoke extraction with solid screen in urban road tunnel fires
Source: PLoS One. 2026 Feb 17;21(2):e0342557. doi: 10.1371/journal.pone.0342557 (PMC12912539; doi:10.1371/journal.pone.0342557)
Supplement: Attachment — Submitted filename: Response to Reviewers.docx [file pone.0342557.s003.docx]

**Response to Reviewers for Manuscript**

Dear Editor,

We sincerely thank you for handling the revision of our manuscript entitled "Study on modular smoke extraction with solid screen in urban road tunnel fires" (Manuscript ID: [PONE-D-25-37621]) and for forwarding the highly constructive and detailed comments from the two reviewers. We are truly grateful to the reviewers for their valuable time and effort. Their insightful and professional comments have been instrumental in significantly enhancing the depth of this research and the clarity of its presentation.

We have given the most serious consideration to every comment and have provided comprehensive, point-by-point responses and revisions in the revised manuscript. The scope of these revisions spans several critical aspects, including clarifying the study's core contributions, strengthening the validation of the numerical model, providing a deeper physical interpretation of the key findings, as well as optimizing figures and refining the language.

We are confident that this thorough revision has substantially improved the scientific quality and academic value of the manuscript. Attached please find our detailed point-by-point response letter and the revised manuscript with all changes highlighted.

We once again extend our gratitude to the editorial office and the reviewers for their invaluable guidance. We respectfully request your consideration of the revised manuscript for the next stage of the publication process.

Our specific, point-by-point responses to each reviewer's comments are detailed below:

**Reviewer #1**

We thank Reviewer #1 for the positive evaluation and the constructive suggestions, which have helped us improve the manuscript. Our point-by-point responses are provided below.

**1. Please, convert this section " Based on the above analysis, a series of numerical..." to the contribution section.**

Thank you for the constructive feedback. We have carefully revised the manuscript according to your suggestions. Specifically, in response to the comment regarding the contribution statement, we have converted the final paragraph of the Introduction section into a clear contribution summary and this change has been tracked and shown in the revised manuscript using the “Track Changes” mode in Microsoft Word..

The original text concluding the Introduction has been replaced with the following paragraph, which now explicitly states the study's objectives and primary contributions in a concise and formal manner:

"Building upon existing research, this study undertakes a systematic numerical investigation using high-fidelity Computational Fluid Dynamics (CFD) to evaluate a novel modular smoke extraction system with solid screens in a full-scale urban road tunnel. The primary contributions are threefold: (1) establishing a parametric framework to quantify the combined effects of key design variables on global extraction efficiency; (2) identifying optimal design thresholds that balance performance with practical cost; and (3) proposing specific engineering guidelines, including an unbalanced airflow strategy, for system optimization. These outcomes provide direct, actionable insights for the design of advanced transverse ventilation systems in urban tunnels."

We believe this revision strengthens the manuscript by clearly framing its value at the outset. We are grateful for the guidance provided and hope the revised text meets the journal's standards.

**2. Authors should include a Flowchart of the problem and the proposed solution**

We sincerely thank the reviewer for this valuable and constructive suggestion. Following your recommendation, we have added a clear and concise Flowchart in the revised manuscript to visually outline the research framework. The Flowchart is presented in Fig.1

Fig.1. Flowchart of the research framework

The flowchart systematically illustrates the logical flow of this study:

**Research Objective:** To achieve global optimization of smoke extraction in urban road tunnels.

**Model Setup:** The establishment of the CFD model, including tunnel geometry and the configuration of the modular exhaust system with solid screens.

**Performance Evaluation Metrics:** The key indicators (e.g., extraction efficiency, CO concentration) used to assess system performance.

**Optimal Design Parameters:** The primary outcomes, identifying the optimal values for critical design variables.

This visual summary enhances the readability and structural clarity of the paper, allowing readers to quickly grasp the problem addressed, the methodology employed, and the solutions derived. We appreciate this insightful suggestion to improve our manuscript's presentation.

**3. Please support the abstract by obtained results.**

We sincerely thank you for this valuable suggestion. We agree that strengthening the abstract with more concrete findings enhances the clarity and impact of our work.

Accordingly, we have thoroughly revised the abstract to better integrate and highlight the key results obtained from our numerical simulations. The revised abstract now more explicitly presents the specific design thresholds and optimal strategies identified in the study, thereby providing stronger support for the conclusions. The updated text is as follows:

“A series of numerical simulations were conducted to evaluate and optimize a modular smoke extraction system integrated with solid screens for urban road tunnel fires. The study aimed to identify key design parameters and propose optimal strategies for global smoke management. The results show that smoke screen height is a critical factor, with a threshold of 1.5 m (25% of tunnel height) significantly enhancing extraction efficiency and reducing high-temperature zones, while vent-screen distance has minimal impact. Dividing the tunnel into five modular zones achieves the optimal balance between hazard control and system cost. Furthermore, an unbalanced airflow distribution strategy—allocating 35% of the total exhaust volume (180 m³/s) to proximate vents and 15% to distal vents—proves most effective in preventing plug-holing and maximizing CO extraction efficiency. These findings provide specific design thresholds and actionable strategies for the optimization of transverse smoke extraction systems in urban road tunnels.”

This revised version has been updated in the manuscript. We believe these modifications have significantly strengthened the abstract. We are grateful for your insightful comment, which has helped improve the quality of our paper.

**4. It could be interesting to summarize the commented literature works in a table to have a clear comparison between all. This could also help precisely formulating the contribution of the paper with respect to previous works.**

We sincerely thank the reviewer for the valuable comment. The suggestion to summarize the commented literature in a table for clearer comparison and to help formulate the paper's contribution is very helpful.

Following this suggestion, we have added a summary table, as shown in Table 1. to the Introduction section of the revised manuscript.

Table 1. Summary of literature on mechanical smoke control in tunnel fires.

| **Ref.** | **Method** | **Core Research Focus** |
| --- | --- | --- |
| [1] | Review | Overview of tunnel fire safety design, including smoke control. |
| [2] | Review | Foundational small-scale duct experiments on fire spread and ventilation. |
| [3] | Review | Fire hazards and mitigation strategies in transportation infrastructures. |
| [4] | Statistical Analysis | Analysis of tunnel fire incident statistics (2012-2023). |
| [5] | Experiment & Theory | Smoke control strategy and design criterion for "complete smoke extraction". |
| [6] | Review | Comparison of longitudinal and transverse ventilation systems for different tunnel types. |
| [7] | Experiment | Comparison of longitudinal vs. transverse ventilation effectiveness in a UTLT. |
| [8] | Experiment & Simulation | Smoke stratification length under longitudinal ventilation. |
| [9] | Theory & Simulation | Quantitative evaluation and optimization of exhaust under lateral centralized mode. |
| [10] | Simulation/Experiment | Influence of vent number/layout on full transverse exhaust performance. |
| [11] | Simulation | Smoke control with different exhaust patterns and air supply in ultra-wide tunnels. |
| [12] | Simulation | Effect of lateral extraction on transverse temperature distribution under ceiling. |
| [13] | Simulation | Mechanical smoke extraction efficiency of multiple lateral vents in an immersed tunnel. |
| [14] | Experiment | Impact of transverse ventilation on smoke spread in an urban tunnel. |
| [15] | Theory & Simulation | Heat and smoke exhaust performance under lateral centralized mode. |
| [16] | Simulation | Compartmentalization and smoke control using Water Screens (WS). |
| [17] | Simulation | Optimal placement and height of solid curtains near exhaust vents. |
| [18] | Experiment | Damper shape and position for smoke confinement under transverse ventilation. |
| [19] | Simulation | A novel inclined smoke screen for lateral extraction in an immersed tunnel. |
| [20] | Simulation | Solid screen enhancement for shaft extraction in urban road tunnels. |

This table directly addresses your point by providing a clear overview of the key literature discussed, which helps to contextualize and precisely frame the contributions of our present work.

Thank you again for this constructive feedback.

**5. Add a comparison between your approach and findings against the recent literature approaches and findings.**

We sincerely thank you for the valuable suggestion. We fully agree that deepening the physical interpretation of our findings and placing them in a broader research context greatly strengthens the discussion.

Following your guidance, we have thoroughly revised the Discussion section to substantially enhance the theoretical explanation of our key results. The main improvements are reflected in the following two aspects:

Enhanced Comparison and Mechanistic Analysis: We have not only added a detailed comparison of our results with previous studies but, more importantly, elucidated the fundamental physical mechanisms (forced pressure difference vs. thermal buoyancy) behind the differing sensitivities of key parameters in mechanical and natural ventilation systems. This places our findings within a clearer framework of disciplinary understanding.

Deepened Interpretation of Physical Mechanisms: For the threshold effect of "smoke screen height," we moved beyond phenomenological description to provide an in-depth explanation from the perspectives of fluid mechanics mechanisms such as "flow separation," "formation of a recirculation vortex zone," and "dynamic smoke reservoir." This clarifies the essential reason for the performance surge and engages in a comparative and mechanistic discussion with existing empirical guidelines.

We have revised the Discussion section based on the above principles. Some of the key modifications are presented below:

**Original Text:** Fig 5 further reveals that increasing the vent-screen distance (d) from 1 m to 3 m has negligible impact on exhaust efficiency, indicating d is a secondary factor within this range.

**Revised Text:** Fig 5 further shows that increasing the vent-screen distance (d) from 1 m to 3 m has a negligible impact on smoke exhaust efficiency, indicating that d is a secondary factor within this range. This finding contrasts with that of Zhang et al. (2022) for a natural ventilation system. In their study, exhaust efficiency was highly sensitive to shaft-screen distance over a short range (0–2.1 m). This discrepancy likely stems from the different driving mechanisms involved: natural ventilation relies primarily on thermal buoyancy, where even minor distance variations can alter the natural convergence path of hot smoke toward the shaft. In contrast, the mechanical transverse exhaust system employed here utilizes fan-induced forced pressure differences, which create an effective suction field across a wider region behind the screen (1–3 m). Consequently, capture efficiency is less sensitive to precise vent-screen spacing within this range.

These revisions aim to make the discussion more insightful and universally significant. Further corresponding refinements have also been made throughout the revised manuscript.

Once again, we are grateful for your insightful comment, which has significantly improved the quality of our paper.

**Reviewer #2**

We are grateful to Reviewer #2 for the thorough review and the insightful comments, which have significantly strengthened the paper. Our responses follow.

**1.** **The CFD modeling section lacks validation of the simulation setup with experimental or previously published benchmark results.**

Thank you for your critical comment regarding the need for validation of the CFD model setup. We fully agree on the importance of validation for ensuring the reliability of simulation results. In response to your suggestion, we have substantially strengthened the validation section in the revised manuscript. Our validation efforts primarily include the following two aspects:

(1) Determination of Model Parameters Based on Established Research

The key parameters for the Large Eddy Simulation (LES), specifically the subgrid-scale model coefficients (Smagorinsky constant Cs=0.18, turbulent Prandtl number Pr=0.5, and turbulent Schmidt number Sc=0.5) were rigorously selected based on authoritative studies concerning buoyancy-driven flows in enclosed spaces. A detailed theoretical justification and literature support for this parameter selection have been comprehensively addressed in our response to Reviewer 2’s Comment #5. This ensures the physical soundness and reliability of our turbulence modeling approach.

(2) Quantitative Validation Against Published Experimental Temperature Data:

To further validate our overall simulation setup, we conducted a dedicated benchmark simulation. We numerically reconstructed the experimental scenario reported by Luo et al. (2013) in Safety Science, which investigated smoke confinement and exhaust efficiency using a modified Opposite Double-Jet Air Curtain. For quantitative comparison, thermocouples were placed in the simulation at locations strictly corresponding to the measurement points specified in the original experimental setup, and temperature data were extracted. The comparative results are presented in Fig 1.

**Fig 1. Model validation against experimental temperature data.**

From the Fig 1, the comparison shows good agreement between our numerical results and the experimental data, confirming the reliability of our model in predicting the thermodynamic behavior of smoke. A new subsection detailing this benchmark validation has been added to the “Methods” chapter (Section “Model construction”) in the revised manuscript, along with a comparative figure. We believe these additions adequately address your concern and firmly establish the credibility of our simulation methodology.

Thank you again for prompting this important improvement to our work.

**2. The boundary conditions are oversimplified, both tunnel ends are treated as open boundaries, but real tunnels often have semi-enclosed conditions or ventilation shafts.**

Thank you for your insightful comment regarding the simplification of boundary conditions in our model. We appreciate your emphasis on the complexity of real tunnel environments, such as semi-enclosed portals or ventilation shafts.

In response to your concern, we would like to clarify the rationale behind our modeling choice from the following perspectives:

(1) Focus on Preliminary Feasibility and Mechanism Isolation

Our current study represents a preliminary investigation into the feasibility of a novel modular smoke control concept. At this initial stage, our primary objective is to explore whether this modular approach can function as a viable smoke management strategy, focusing on key design variables such as solid screen height, zone partitioning, and exhaust airflow distribution. The use of open boundaries at both tunnel ends establishes a benchmark, controlled flow environment for this feasibility assessment. This simplified configuration intentionally isolates the proposed system from external complexities such as portal effects or coupled shaft ventilation, allowing for a clearer preliminary evaluation of whether the modular concept demonstrates fundamental viability under controlled conditions.

(2) Common Practice in Foundational and Parametric Studies

This modeling approach is well-established in foundational research focused on comparing ventilation or extraction system performance under controlled conditions. For instance:

- Gao et al. (2012), in their study proposing an opposite-double air curtain ventilation system for creating smoke-free evacuation passageways, employed a simplified tunnel model with open boundaries to isolate and evaluate the core performance of the novel ventilation strategy against traditional systems.
- Xu et al. (2022), in their investigation of heat and smoke exhaust efficiency under lateral centralized mode, similarly adopted open boundary conditions to focus on the quantitative evaluation of exhaust system performance and the determination of reasonable exhaust volume, excluding complex portal or shaft interactions.
- Li et al. (2022), in their simulation study on smoke control in ultra-wide tunnels, utilized a model with both ends open to compare top and sidewall exhaust patterns.
- Zhang et al. (2022), while investigating the effect of solid screens on vertical shaft performance, also employed open boundaries to concentrate on the screen-shaft interaction dynamics.

These precedents confirm that such a simplification is a valid and accepted methodology for parametric studies aimed at deriving comparative insights and fundamental trends.

We fully acknowledge that real tunnel geometries, including semi-enclosed portals and ventilation networks, will influence factors like longitudinal pressure distribution and overall smoke confinement. Our current model does not capture these specific effects. Therefore, as a direct follow-up to this foundational work, we plan to conduct a detailed subsequent study. This future work will incorporate realistic portal conditions and auxiliary ventilation shafts to comprehensively evaluate the system’s performance in more complex, practical scenarios. To enhance transparency and methodological rigor, we will explicitly include the assumption of open boundaries as part of the model setup description in the “Methods” section. Furthermore, to ensure methodological transparency, we have explicitly stated the simplifying assumptions regarding boundary conditions (modeling both portals as open boundaries and not considering additional ventilation shafts) and their implications in the newly added section "Model assumptions and limitations" of the revised manuscript. The investigation of their influence is thereby positioned as a clear direction for future research.

**3. The fire source description (n-heptane pool, 20 MW) is not supported by experimental verification or heat release rate time curve; assuming a steady HRR neglects realistic transient behavior.**

Thank you for your valuable comments regarding the fire source description and HRR modeling. We appreciate your insights, which have helped us clarify and strengthen the methodological rationale of our study. In response to your concerns, we would like to explain our approach from the following three perspectives:

(1) Focus on System Performance Rather than Fire Dynamics

The primary objective of this study is to evaluate the effectiveness of modular mechanical smoke extraction systems, not to reproduce detailed transient fire dynamics. We adopted a simplified fire source model to clearly analyze the influence of key system parameters—such as screen height, zoning, and airflow distribution—without the additional complexity introduced by transient fire growth. This approach is common in performance-oriented ventilation studies.

(2) Use of a Conservative, Worst-Case HRR Scenario

To ensure the applicability of our findings to high-risk scenarios, we selected a steady-state HRR of 20 MW, corresponding to a severe fire involving heavy goods vehicles in road tunnels. This represents a conservative; worst-case design fire commonly used in tunnel safety assessments. Evaluating system performance under this sustained severe condition enhances the reliability and safety relevance of our conclusions.

(3) Widespread Acceptance in Comparable Studies

The use of a steady HRR with representative fuels such as n-heptane is well-established in tunnel fire research focused on ventilation and smoke control. For example:

- Zhang et al. (2022) adopted a steady 5–10 MW heptane fire to study vertical shaft extraction.
- Chen et al. (2024) used a constant HRR in their evacuation simulation in a naturally ventilated tunnel.
- Li et al. (2022) also employed a steady 20 MW fire to evaluate smoke control in ultra-wide tunnels.

These precedents demonstrate that a steady HRR is a valid and widely accepted simplification for comparative analysis of smoke management systems.

We acknowledge that real fires exhibit transient growth, and such dynamics could be considered in future studies focusing on time-dependent evacuation or detection. In the revised manuscript, we will clarify the rationale for using a steady HRR and cite the aforementioned studies to support our methodological choice.

To further demonstrate the rigor of our study and address your concern, if you consider it necessary to validate the findings under transient fire conditions, we commit to performing additional simulations in subsequent work and sharing the relevant data with you.

Thank you again for your constructive feedback, which has helped improve the clarity and rigor of our work.

**4. The mesh independence study is only justified by the D* criterion but not actually demonstrated through sensitivity analysis. The accuracy of results at 0.25-0.5 m mesh remains questionable.**

Thank you for your valuable comment regarding the justification of our mesh independence study. We fully agree that relying solely on the D* criterion as a theoretical basis is insufficient, and actual sensitivity analysis is required to demonstrate the accuracy of the chosen mesh. In direct response to this point, we have conducted a crucial supplementary investigation.

The specific revisions and supplementary analysis are as follows:

Designing around the reference grid size (0.1D* ≈ 0.35 m) calculated from the original Equation (1), we tested three grid schemes:

Case M1(Fine): 0.125 m in the fire region, 0.25 m elsewhere.

Case M2 (Base): 0.25 m in the fire region, 0.5 m elsewhere (the scheme ultimately adopted in the original manuscript).

Case M3 (Coarse): 0.5 m in the fire region, 1.0 m elsewhere.

We selected temperature as the monitoring variable, given its critical importance for evaluating smoke extraction performance. A monitor was placed 1 m directly below the center of Vent III (Z=5.0 m), a key location prone to plug-holing. The newly added grid independence plot (Fig. 2) shows the temperature evolution at this point:

**Fig 2. Temperature-time profiles from the mesh sensitivity analysis.**

Overall, the three Cases predict broadly consistent temperature trends. Case M3 exhibits relatively greater fluctuations in its predicted curve after stabilization. The results from Case M1 and Case M2 are more consistent with each other. However, the computational cost of Case M1 is over 10 times that of Case M2, making it computationally inefficient. Based on this analysis, Case M2 (0.25m in fire region / 0.5m elsewhere) achieves the optimal balance between accuracy and efficiency. It reliably captures the critical temperature field characteristics near the exhaust vent that affect safety assessments while avoiding excessive computational burden. Therefore, we are confident that the conclusions regarding flow fields, temperature distributions, and extraction efficiency drawn using this grid Case are reliable and robust.

We have added the detailed process of the grid independence study, the results plot (Fig. 2), and related discussions to the “Methods” section of the manuscript. We appreciate your valuable feedback, which has significantly strengthened the methodological rigor of our work.

**5. The study does not include any uncertainty analysis or sensitivity study on turbulence model parameters?**

Thank you for your valuable comment regarding the uncertainty analysis of turbulence model parameters. We have carefully considered this important point.

As you are aware, the selection of subgrid-scale model parameters in Large Eddy Simulation (LES)—such as the Smagorinsky constant (Cs), turbulent Prandtl number (Pr), and turbulent Schmidt number (Sc)—can indeed influence computational accuracy. In our original manuscript, we did not provide sufficient justification for the selection of these three parameters, and we sincerely apologize for any confusion this may have caused. To ensure the reliability of our simulation results, these parameters were not chosen arbitrarily. Instead, they were determined strictly based on established authoritative research in fire science and computational fluid dynamics concerning strong buoyancy-driven flows in enclosed spaces.

Specifically, our parameter selection (Cs=0.18, Pr=0.5, Sc =0.5) is supported by the following key references:

The choice of Cs=0.18 is supported by the work of Zhang et al. (2002), who validated LES for fire-induced buoyant flows in a compartment and found this value to yield good agreement with experimental turbulence statistics.

The values Pr =0.5 and Sc=0.5 are the default and widely validated settings recommended in the FDS Technical Reference Guide for fire and smoke simulations, ensuring physically consistent turbulent transport of heat and species.

Furthermore, the validity of this parameter set (Cs=0.18, Pr=0.5, Sc=0.5) is directly evidenced by the successful numerical reconstruction of LUO et al.'s study. As detailed in our response to Comment #1, the simulated temperature fields showed good agreement with the experimental measurements (see Fig X).

Thus, our selection is grounded in literature specifically relevant to strong buoyancy flows and in the standard practice of a well-validated fire simulation tool. We believe this approach provides a reliable basis for the conclusions drawn in our study.

We will add a brief justification referencing Zhang et al. (2002) and the FDS guide in the “Methods” section of the revised manuscript to enhance clarity.

Thank you again for your insightful suggestion.

**6.The governing equations and numerical schemes of FDS are briefly cited but not adequately described or justified for the chosen grid scale and fire size.**

Thank you for your important comments regarding the numerical methodology section. We fully agree that thorough justification of model parameters and grid scale is fundamental to ensuring the reliability of the results. In response to your suggestions, we have made significant additions to the “Methods” section of the manuscript, as detailed below:

(1) Detailed Justification of Key Subgrid-Scale Model Parameters

We have added a detailed discussion and justification for the key parameters of the Large Eddy Simulation (LES) subgrid-scale model in the “Methods” chapter. This specifically includes the Smagorinsky constant (Cs), the turbulent Prandtl number (Pr), and the turbulent Schmidt number (Sc). The values of these parameters (Cs= 0.18, Pr = 0.5, Sc = 0.5) were not chosen arbitrarily. They were rigorously determined based on authoritative studies concerning buoyancy-driven flows in enclosed spaces (e.g., Zhang et al., 2002) and the recommendations of the FDS Technical Reference Guide. To validate the reasonableness of this parameter set, we also performed a numerical reconstruction and benchmarking against a previously published experimental study (the specifics of which are detailed in our response to Comment 1). This ensures that our turbulence model is physically sound and capable of accurately capturing the interaction between fire smoke and the extraction system.

(2) Grid Independence Analysis Based on Characteristic Fire Scale

We have added a dedicated subsection on “Grid Resolution Analysis” within the “Methods” chapter. Following established practices in tunnel fire CFD research, we have justified the grid size based on the characteristic fire diameter (D*). Specifically, we calculated the characteristic diameter $D^{*}$ for the 20 MW fire source. Citing widely accepted criteria in the field (e.g., Drysdale, 2011), we argue that simulation results can be considered grid-independent and reliable when the grid size is smaller than $0.1D^{*}$. Our final grid configuration (refined to 0.25 m in the fire source region and 0.50 m elsewhere) fully satisfies this requirement. The detailed formulas, calculation process, and conclusions are now fully presented in the manuscript (the core arguments are summarized in our response to Comment 4).

Through these additions, we aim to make the description of the numerical methodology more complete, transparent, and aligned with best practices in the field. These revisions directly address your core concerns regarding the “brief citation of governing equations and schemes” and the “inadequate justification for the chosen grid scale and fire size.”

Thank you again for your valuable feedback, which has significantly enhanced the methodological rigor of our study.

**7. The choice of CO₂ as a tracer for smoke extraction efficiency may not accurately represent smoke behavior; direct smoke or soot mass fraction analysis would be more relevant.**

Thank you for your valuable feedback. We fully understand and appreciate your rigorous consideration regarding the choice of smoke tracer. Your suggestion to directly analyze soot mass fraction is constructive and would contribute to a more comprehensive assessment of personnel safety conditions.

Considering the primary objective of this study—to compare the engineering performance of different modular smoke extraction systems—we selected CO₂ as the tracer for calculating smoke extraction efficiency. This decision is based on the following three research considerations and established practices in the field:

(1) Direct use of smoke volume flow makes it difficult to distinguish real smoke from entrained air. The gas discharged from exhaust vents is actually a mixture of smoke and air. Using the total volume to calculate efficiency would significantly overestimate system performance. In contrast, the mass flow rate of CO₂ effectively represents the transport of "real smoke," avoiding interference from air entrainment. This method has been widely adopted and recognized in the field. For example, Li et al. (2022) used CO₂ mass flux to calculate efficiency when evaluating smoke exhaust patterns in ultra-wide tunnels, and Zhang et al. (2022) similarly used CO mass flow as an evaluation metric in their study on shaft smoke extraction. This reflects the standardization and comparability of the approach.

(2) Regarding the suggestion to use soot mass fraction, it is noteworthy that soot transport involves complex processes like sedimentation and deposition. These processes introduce significant uncertainty into its mass flow calculations in physical models, rendering soot less suitable for robust comparisons of different system configurations. In contrast, CO₂ is a gaseous product whose transport simulation is more stable. This stability makes CO₂ more appropriate for the equitable evaluation of mechanical performance across systems.

To address your concerns more comprehensively, we will add a clarification in the Methods section of the revised manuscript that explains the rationale for using CO₂ as a key indicator.

**8.** **The assumption that CO concentration is a surrogate for toxicity and evacuation safety is too simplified; combined effects of CO, temperature, and visibility should be considered.**

Thank you very much for your valuable comment regarding the need to consider the combined effects of CO, temperature, and visibility for safety assessment. We fully agree that using CO concentration as a sole safety indicator is an oversimplification. In response to your concern, we have made significant additions to the revised manuscript.

The main revisions are as follows:

Supplemented Temperature Field Analysis: In addition to the original CO concentration analysis, we have now simulated the temperature field on the central longitudinal plane for different zoning scenarios (Z3, Z5, Z7, Z9). In all temperature contours, we have prominently marked the 68°C isotherm with a bold red line (a temperature widely recognized as the threshold for instantaneous human burns and a sharp decline in evacuation capability). The simulated results are presented in Fig. 3. This additional temperature analysis serves as a crucial supplement and further verification of the original CO-based assessment.

**(a)**

**(b)**

**(c)**

**(d)**

**Fig 3. Smoke temperature distribution in central surface with varying numbers of modular zones: (a)** **3 zones; (b) 5 zones; (c)** **7 zones; (d) 9 zones;**

Integrated Assessment Results: Combining the new temperature fields with the original CO distribution, we conducted a comprehensive evaluation:

Z3 (3 zones): As shown in the original Fig. 5b, it has the longest severe CO hazard zone (132 m). The newly added temperature contour (Fig. 3) reveals that its high-temperature area above 68°C is also the most extensive, consistent with the CO assessment, indicating that the Z3 scheme is also poor at controlling thermal hazards.

Z5 (5 zones) & Z7 (7 zones): Both outperform Z3 in CO control. The new temperature contours further confirm that the high-temperature influence ranges of both schemes are significantly reduced, demonstrating excellent safety performance. However, from an economic perspective for engineering applications, the Z5 scheme (5 zones) entails significantly lower costs for equipment, installation, and maintenance compared to the Z7 scheme (7 zones). Therefore, while achieving comparable safety performance, the Z5 scheme offers superior cost-effectiveness.

Z9 (9 zones): Although it has a slight advantage in controlling moderate CO hazard zones, the temperature contour reveals an important phenomenon: its 68°C high-temperature area is larger than that of Z5 and Z7. This is likely due to excessive zoning leading to insufficient airflow per exhaust vent, failing to effectively cool and remove the high-temperature smoke plume near the fire source.

Strengthened Conclusion: By comprehensively considering the two most critical life safety factors—CO toxicity (original analysis) and high-temperature thermal hazard (new analysis)—the advantage of the five-zone (Z5) scheme becomes even more prominent. It not only performs best in controlling toxic gas diffusion (original conclusion) but also achieves the optimal balance in suppressing high-temperature smoke spread and reducing thermal exposure risk (new evidence). Simultaneously, its number of zones represents the optimal trade-off between safety and economy. Therefore, our recommendation of Z5 as the optimal scheme is now supported by dual verification, making it more robust.

We have updated the relevant section (Results and Discussion) in the manuscript, including the new temperature contours and analysis. Thank you again for prompting us to improve the safety assessment dimension of this research.9. The study claims an optimal smoke screen height of 1.5 m, but this conclusion appears case-specific. No scaling or generalization method is discussed for tunnels with different geometries or fire sizes.

**9. The study claims an optimal smoke screen height of 1.5 m, but this conclusion appears case-specific. No scaling or generalization method is discussed for tunnels with different geometries or fire sizes.**

Thank you for your valuable comment regarding the generalizability of our findings. We fully agree that tunnel fire safety design should consider variations in geometrical dimensions and fire scenarios. Regarding your concern that our conclusions may be "case-specific," we would like to clarify the following:

The tunnel cross-section dimensions adopted in this study (width 10 m × height 6 m) adhere to the Chinese national standard "Code for Design of Road Tunnels" (JTG D70-2018). The code specifies a minimum vertical clearance of 5.0 m for vehicular tunnels, and the 6 m height includes the structural space required for ventilation ducts, lighting, and smoke exhaust equipment, which is very common in actual urban road tunnels. The width of 10 m corresponds to a typical bidirectional four-lane urban road tunnel (each lane 3.5 m wide with 0.75 m shoulders on both sides). This cross-section is widely used in Chinese urban tunnels.

It is worth noting that this cross-section size has been widely adopted in tunnel fire research. For instance, Ji et al. (2013) used a 10 m × 5 m cross-section, Seike et al. (2019) employed 10 m × 7 m, and Zhang et al. (2022) investigated a full-scale urban road tunnel with a 10 m (width) × 5.2 m (height) cross-section. Therefore, the 10 m × 6 m cross-section used in our study represents a typical bidirectional four-lane urban tunnel in China, demonstrating its representativeness in both engineering practice and academic research. Findings based on this typical cross-section can provide a reference for designing smoke control systems in urban tunnels in China. Our results suggest that, under the adopted typical tunnel cross-section, a smoke screen height exceeding 1.5 m may lead to a relatively significant improvement in smoke control effectiveness, offering practical guidance for similar engineering projects.

We fully acknowledge that variations in tunnel cross-section dimensions may affect smoke control performance. The scope of applicability of this study, particularly regarding the selected typical tunnel cross-section, has been clearly stated in the newly added section **"Model assumptions and limitations"** to enhance the clarity and practical relevance of the conclusions. In future work, we will systematically conduct parametric studies on tunnels of different dimensions and under various fire scenarios to further verify the boundaries of applicability of our conclusions and explore design generalization methods based on proportional relationships.

Thank you again for your careful review and valuable suggestions. We will further refine the relevant content in the revised manuscript and look forward to your continued guidance.

**10. The selection of five modular zones as optimal lacks a quantitative cost-benefit analysis or an objective optimization framework; it relies solely on limited simulation scenarios.**

Thank you very much for your insightful comment regarding the need for a quantitative optimization framework to support the selection of the five-zone configuration. We fully agree that an objective, quantifiable decision-making process is essential. In direct response to your valuable suggestion, we have substantially revised the manuscript by developing and applying a new quantified safety-benefit-to-cost decision model.

(1). Model Development

The core idea is to define a single metric that comprehensively reflects both the safety performance and the system complexity cost of each configuration. We propose the Benefit Index, *B*_i_, defined as:

$$B_{i}=\frac{S_{i}}{C_{i}}$$

Where:

*S*_i_ (Safety Benefit): Quantifies the total safety improvement of configuration i compared to the most hazardous baseline scenario. A higher *S*_i_ indicates greater absolute safety improvement.

$$S_{i}=\alpha\left(L_{s,\max}-L_{s,i}\right)+\beta\left(L_{m,\max}-L_{m,i}\right)$$

*L*_s,max_ and *L*_m,max_ are the maximum lengths of the severe and moderate hazard zones, respectively, among all configurations. α and β are weighting coefficients (set to α=0.8 and β=0.2) to reflect the higher priority of controlling severe hazards.

*C*_i_ (Cost): Represented directly by the number of zones, *N*_i_ .More zones imply greater system complexity and typically higher costs for equipment, control, and maintenance. A higher *C*_i_ indicates a higher cost.

$$C_{i}=N_{i}$$

Thus, the physical meaning of *B*_i_ is "the safety benefit per unit zone," or the "safety cost-effectiveness" of a configuration. A higher *B*_i_ value signifies a better balance between safety and cost.

(2). Model Application and Results

Applying this model to the data from the original Fig. 5 yields the following results:

Table 1. Comparison of the Benefit Index (B_i_) for different zoning configurations.

| Case No. | Safety Benefit *S*_i_ | Cost *C*_i_ | Benefit Index *B*_i_ |
| --- | --- | --- | --- |
| Z3 (3 zones) | 0.0 | 3 | 0.00 |
| Z5 (5 zones) | 58.6 | 5 | 11.72 |
| Z7 (7 zones) | 24.2 | 7 | 3.46 |
| Z9 (9 zones) | 20.6 | 9 | 2.29 |

The results clearly demonstrate: The five-zone configuration (Z5) achieves a significantly higher Benefit Index than all other alternatives, which indicates that the Z5 configuration delivers the highest safety return per unit of system complexity.

By introducing this quantitative decision model, the selection of the optimal number of zones is transformed into a clear optimization problem: identifying the configuration with the maximum Benefit Index Bi. The model's output provides objective and robust evidence that the five-zone (Z5) configuration is the optimal solution.

We have integrated this complete optimization framework, including the formulas, calculations, and conclusions, into the "Results and Discussion" section of the revised manuscript. We sincerely appreciate your constructive feedback, which has greatly enhanced the rigor and persuasiveness of our study's conclusions.

**11. The influence of longitudinal air movement, natural draught, and vehicle-induced flow has been neglected?**

Thank you for raising the important issue regarding the neglect of longitudinal air movement, natural draught, and vehicle-induced flow in our study. Your point is very insightful, and we fully recognize the importance of these factors in real tunnel environments. The omission of these influences in our current model indeed represents a limitation of this work. We also acknowledge that the original manuscript’s failure to explicitly state this simplifying assumption affected the rigor of the paper, and we sincerely apologize for this oversight.

The primary objective of this study was to conduct a preliminary exploration and proof-of-concept for the technical approach of “modular smoke extraction systems integrated with solid screens.” To more clearly focus on the initial trends in the influence of the system’s core parameters (such as screen height, number of zones, etc.), we consciously controlled variables in the modeling process and temporarily did not introduce complex external conditions such as longitudinal ventilation, natural draught, and vehicle flow.

we fully recognize that these practical environmental factors can interact with the smoke extraction system in complex ways, and their influence could be significant. Incorporating them into the model would elevate the complexity of the problem to a level requiring independent, systematic investigation, which is beyond the scope of this preliminary exploration. Therefore, to prevent the analysis from becoming overly complex and to ensure a clear research focus, we adopted this simplifying assumption. Accordingly, we have explicitly stated in the opening of the revised “Methods” section that this study temporarily does not consider these external factors, thereby enhancing the transparency of the research.

Once again, we sincerely appreciate your valuable insights. Your comments have helped us more accurately define the scope and value of the current work and have provided a clear path for our subsequent research.

**12.** **The analysis of the plug-holing phenomenon is largely qualitative; no flow field visualization or mass balance is provided to substantiate the mechanism.**

Thank you for your insightful comment that our analysis of the plug-holing phenomenon could be more in-depth. We fully agree that clearer visualization of the flow field would be beneficial to substantiate the mechanism. In response, we have conducted a crucial supplementary analysis in the revised manuscript.

The main revisions and findings are as follows:

(1).Supplemented Temperature Field Visualization on a Key Plane: To more directly observe the flow structure near the exhaust vents, we have added a new horizontal temperature slice. This slice is positioned at a height of 0.5 m below the tunnel ceiling (Z = 5.5 m), which is a key plane for visually assessing whether cool air is being entrained. We performed additional simulations for the four key scenarios from the original Fig. 8 to obtain more detailed visual evidence. The simulated results are presented in Fig. 4.

|   **(a)** |   **(b)** |
| --- | --- |
|   **(c)** |   **(d)** |

**Fig 4. Temperature distributions on the horizontal plane at Z = 5.5 m:** (a) Vent III at 30%; (b) Vent III at 45%; (c) Vent IV at 5%; (d) Vent IV at 20%.

(2).Provided Clearer Visual Verification: Based on the new temperature slice (new Fig. 4), we obtained a clearer picture of the flow:

- In scenarios where plug-holing did NOT occur (i.e., the recommended Vent III at 30% and Vent IV at 5%), the temperature distribution around the vent area is continuous and uniform with the surrounding hot smoke, showing no significant ingress of cool air. This indicates the vent is primarily extracting smoke from the upper hot layer.
- In scenarios where plug-holing DID occur (i.e., Vent III at 45% and Vent IV at 20%), a distinct, isolated low-temperature zone can be clearly observed directly beneath the exhaust vent. This zone presents a sharp visual contrast with the surrounding hot smoke, directly illustrating the process where cool air from below is "short-circuited" into the vent.

(3).Strengthened Mechanism Substantiation: This supplementary visualization makes the core mechanism of "cool air ingress" more explicit and intuitive. The isolated low-temperature zone beneath the vent provides direct visual evidence of the flow field for the plug-holing phenomenon. It strongly supports the original conclusion that an optimal range exists for airflow distribution (e.g., 30% and 5%), beyond which plug-holing is induced.

We have integrated this key temperature slice (Fig. 4) and its analysis into the "Results and Discussion" section. We sincerely appreciate your valuable feedback, which prompted us to provide more comprehensive visual verification of the plug-holing phenomenon.

**13. The smoke extraction efficiency definition based on CO₂ flux is not fully consistent with standard approaches, and the formula needs clearer explanation and justification.**

We sincerely appreciate the reviewer’s insightful comment regarding the definition and justification of the smoke extraction efficiency based on CO₂ mass flux. We agree that a clear and well-justified methodology is essential for the rigor and reproducibility of the study.

In response, we have further clarified and expanded the explanation in the revised manuscript (in the section “Smoke extraction efficiency,” around Equation (2)). Specifically, we have:

(1) Explicitly stated the rationale for using CO₂ as a tracer:

CO₂ is a major combustion product with a relatively stable and predictable yield under the controlled heptane fire condition. Its mass flow rate serves as a robust and computationally accessible proxy for the total smoke mass flow, avoiding uncertainties associated with direct volumetric measurements in heterogeneous, temperature-varying smoke layers.

(2) Clearly defined each variable in Equation (2):

m_e_: CO₂ mass flow rate exhausted through the vent, (kg/s);

m_z_: CO₂ mass flow rate in the tunnel cross-section upstream of the vent, (kg/s);

η: represents the exhaust efficiency of an exhaust vent (%)

Additionally, to further enhance the clarity and reproducibility of our methodology, we have updated Figure 1c in the revised manuscript (as shown in the updated Fig 5.). The schematic diagram now explicitly labels the specific measurement locations for m_e_ and m_z_. This visual clarification directly maps the variables in Equation (2) to their spatial positions in the simulation setup.

Fig 5. Schematic of CO₂ mass flow rate measurement at an exhaust vent.

(3) Cited established precedents in the literature:

This approach of using a conserved species mass flux ratio to evaluate smoke extraction efficiency is well-grounded in previous tunnel fire studies. For instance:

Zhang et al. (2022, Fire Technology) used CO mass flow rate ratios to assess smoke extraction efficiency in vertical shafts with solid screens.

Li et al. (2022, Fire) calculated smoke extraction efficiency based on the mass flux of CO₂ for ultra-wide tunnel fires.

Yao et al. (2019, International Journal of Thermal Sciences) employed CO₂ mass flow rates to evaluate natural ventilation performance.

Our method follows the same principle and is consistent with these accepted practices in the field.

We believe these revisions provide the necessary clarification and justification, ensuring that the method is transparent, reproducible, and aligned with established research practices.

We are grateful to the reviewer for raising this point, which has allowed us to better situate our methodological choice within the established research framework.

**14. Figures (especially Figs. 2–8) lack proper axis labels, quantitative scales, and legends.**

We sincerely thank you for pointing out the need for improvement in our figures. We agree that clear and well-annotated figures are crucial for readability.

In response, we have carefully revised all figures in the manuscript (Figs. 2–8) to address the issues raised. The revisions focused on ensuring that all axes are properly labeled with clear scales, and that legends are included and made unambiguous where necessary. Below is a representative example comparing a key figure before and after revision:

**Original：**

**Revised:**

**Original：**

**Revised:**

**Original：**

**Revised:**

All such modifications, along with numerous other refinements throughout the text, have been incorporated into the revised manuscript.

We believe these comprehensive edits have significantly improved the clarity and professional presentation of the graphical data in the paper.

We appreciate your constructive feedback, which has helped enhance the overall quality of our work.

**15. The study ignores the impact of varying tunnel slope, which in practice influences smoke stratification and backlayering.**

We sincerely thank the reviewer for raising this insightful and important point regarding the influence of tunnel slope on smoke stratification and back-layering. We fully acknowledge that, in practical engineering, the slope of a tunnel is a critical factor that significantly affects fire-driven smoke flow, and its inclusion would provide a more comprehensive understanding of system performance under real-world conditions.

In the present study, our primary objective was to conduct a preliminary investigation into the feasibility and parametric optimization of the proposed modular smoke extraction system with solid screens in a controlled, baseline configuration. To isolate and clearly elucidate the fundamental effects of key design variables—such as screen height, zone partitioning, and airflow distribution—we adopted a horizontal tunnel model as the initial, simplified scenario. This approach allowed us to establish a clear performance benchmark for the system without the compounding interactions introduced by slope.

We completely agree with the reviewer that the impact of tunnel slope constitutes a vital and complex extension of this work. It indeed warrants a dedicated and systematic analysis. Accordingly, the assumption of a horizontal tunnel and its acknowledgment as a limitation have been explicitly addressed in the newly added section "Model assumptions and limitations". The examination of the interaction between tunnel inclination and the system performance is consequently framed as a focus for subsequent research. We plan to develop specific models to address this question and optimize the system for varied topographic conditions.

Once again, we are grateful for this valuable suggestion, which not only strengthens the discussion of our current work's limitations but also provides a clear and important direction for our future research endeavors.

**16. The discussion section merely restates the results without deeper physical interpretation or comparison with previous numerical or experimental studies.**

Thank you for the insightful comment. We agree that deepening the physical interpretation of our findings and placing them in a broader research context greatly strengthens the discussion. Accordingly, we have thoroughly revised the Discussion section, substantially enhancing the theoretical explanation of our key results. Some of the modifications are listed here, with further changes tracked and shown in the revised manuscript using the “Track Changes” mode.

**Revision 1:**

**Original Text:** Fig 5 further reveals that increasing the vent-screen distance (d) from 1 m to 3 m has negligible impact on exhaust efficiency, indicating d is a secondary factor within this range.

**Revised Text:** Fig 5 further shows that increasing the vent-screen distance (d) from 1 m to 3 m has a negligible impact on smoke exhaust efficiency, indicating that d is a secondary factor within this range. This finding contrasts with that of Zhang et al. (2022) for a natural ventilation system. In their study, exhaust efficiency was highly sensitive to shaft-screen distance over a short range (0–2.1 m). This discrepancy likely stems from the different driving mechanisms involved: natural ventilation relies primarily on thermal buoyancy, where even minor distance variations can alter the natural convergence path of hot smoke toward the shaft. In contrast, the mechanical transverse exhaust system employed here utilizes fan-induced forced pressure differences, which create an effective suction field across a wider region behind the screen (1–3 m). Consequently, capture efficiency is less sensitive to precise vent-screen spacing within this range.

**Revision 2:**

**Original Text:** In contrast, smoke screen height (h) plays a critical role. At h=0.5 m, efficiency remains unchanged versus the no-screen scenario. At h=1 m, modest improvements occur (for vent I, 2.22% for II, 2.91% for III, 6.14% for IV). When h≥1.5 m, efficiency surges by 18% uniformly across all vents. This bifurcation arises because low screens (h ≤ 1 m) only obstruct ceiling-proximal smoke, permitting easy escape beneath the screen with sustained high horizontal velocity. Taller screens (h ≥ 1.5 m) force smoke to traverse longer escape paths, increasing flow resistance and reducing horizontal velocity—thereby enhancing vent capture efficiency.

**Revised Text:** In contrast to the vent-screen distance, the smoke screen height (h) plays a critical role in determining exhaust efficiency. When h = 0.5 m, the efficiency remains almost unchanged compared to the no-screen scenario. A slight improvement is observed at h = 1 m, while a marked efficiency surge occurs once h≥1.5 m. This threshold behavior stems from a fundamental alteration in the smoke flow pattern induced by the screen: A lower screen (h≤1.0 m) provides an insufficient effective blocking area to cause flow separation of the main smoke stream. Consequently, the smoke is only slightly perturbed and bypasses the screen with sustained high horizontal velocity, as shown in Fig. 3a-c. When the screen height increases to 1.5 m (25% of the tunnel height), its effective blocking area becomes adequate to trigger significant flow separation, forming a stable recirculation vortex zone upstream of the screen. This zone corresponds to the extensive "negative velocity zone" observed in Fig. 3d-e. Acting as a dynamic "smoke reservoir," it not only substantially dissipates the horizontal momentum of the smoke but also prolongs its residence time near the exhaust vent, thereby greatly enhancing the capture probability.

The critical screen height identified in this study (25%) aligns well with the effective screen-height ratio range (17%–29% of tunnel height) suggested by Zhang et al. (2022) for natural ventilation shafts, confirming the applicability of this empirical guideline across different systems. Notably, the critical value here is closer to the upper limit of that range. This likely reflects an intrinsic difference between mechanical exhaust and natural ventilation systems: the active suction in mechanical systems exerts a stronger disturbance on the stability of the smoke layer, thus requiring a taller screen to maintain sufficient smoke layer thickness to counteract the enhanced airflow disturbance and ensure efficient smoke accumulation.

**Revision 3:**

**Original Text:** This divergence stems from zone-configuration effects: Z3’s excessive zone length increases vent-fire distance, enlarging smoke dispersion; Z9’s high zone density reduces individual vent airflow, impairing local smoke extraction near the fire.

**Revised Text:** This difference stems from the combined effects of different zone configurations on the "spatial interception density" of smoke and the "local suction intensity" of exhaust vents. In the Z3 configuration, an insufficient number of vents results in excessively large spacings between adjacent vents, creating an extensive "capture blind zone" downstream of the fire. Smoke spreads freely before reaching the first effective vent, leading to thickening of the smoke layer and consequently maximizing the longitudinal pollution range. Conversely, the Z9 configuration overly disperses the total airflow, resulting in significantly insufficient airflow at individual vents. Near the fire source, smoke possesses extremely high thermal buoyancy and horizontal momentum, while the suction force at vents in this region is insufficient to establish a dominant local pressure differential. Consequently, a large amount of high-concentration smoke is transported downstream, leading to a larger severe hazard zone. As smoke propagates downstream, its horizontal momentum decays due to wall friction and air entrainment. The diminished smoke inertia allows the suction effect of distant vents to become relatively more prominent. This coupling effect of "smoke momentum decay" and "relative enhancement of exhaust power" enables effective containment and extraction of downstream smoke, thereby reducing the extent of the moderate hazard zone. The Z5 configuration achieves the optimal balance: a sufficient number of partitions shortens the free diffusion distance of smoke, enabling early and effective interception, while the airflow allocated to each vent (especially those near the fire) generates sufficiently strong local suction to significantly suppress long-distance smoke propagation. As a result, the extents of both severe and moderate hazard zones are effectively controlled.

Further detailed modifications have been made throughout the manuscript, as highlighted in red in the revised version.

We believe these targeted revisions, along with the additional changes marked throughout the manuscript, have fully addressed your constructive suggestion. Thank you again for your valuable feedback, which has undoubtedly improved our paper.

**17. English expression requires improvement; there are frequent grammatical errors and long sentences that reduce readability.**

We sincerely thank you for this important feedback. We fully acknowledge that the clarity and readability of the manuscript are paramount. In direct response to your comment, we have undertaken a thorough, line-by-line revision of the entire manuscript to correct grammatical errors, simplify overly complex sentences, and improve the overall flow and precision of the language.

Some representative examples of these sentence-level revisions are provided below:

**Original Text:** Traditional mechanical smoke extraction methods in urban tunnels primarily include longitudinal and transverse smoke extraction systems.

**Revised Text:** Traditional mechanical smoke extraction in urban tunnels is primarily achieved through two systems: longitudinal and transverse

**Original Text:** Xu et al [15]. studied smoke exhaust performance in tunnel fires under the lateral centralized smoke exhaust mode through theoretical models and FDS simulations and the result show that increasing the exhaust volume improves heat and smoke exhaust efficiency, with optimal performance at a single-sided exhaust volume of 180 m3/s, achieving efficiencies of 54.2% and 47.3% for heat and smoke, respectively.

**Revised Text:** Xu et al. [15] evaluated smoke exhaust performance under a lateral centralized mode, employing both theoretical models and FDS simulations. Their results indicate that increasing the exhaust volume improves the efficiency of both heat and smoke removal. The optimal performance was observed at a single-sided exhaust volume of 180 m³/s, achieving heat and smoke exhaust efficiencies of 54.2% and 47.3%, respectively.

**Revised Text:** However, most existing studies have concentrated on the smoke control of individual or small-scale regions, overlooking the complexities of coordinating extraction across the entire tunnel system.

**Original Text:** However, the focus has predominantly been on localized smoke control in isolated or small-scale sections, often neglecting the challenges of coordinating extraction across the entire tunnel network.

All such modifications, along with numerous other refinements throughout the text, have been incorporated into the revised manuscript.

We believe these extensive edits have significantly enhanced the readability and academic quality of the paper. We are grateful for your careful review and constructive suggestion.
